# Internal structure and factorial invariance of the Patient Health Questionnaire −9 (PHQ-9) in a large Argentinean sample

**DOI:** 10.1186/s12888-026-07820-9

**Published:** 2026-01-28

**Authors:** Pablo Ezequiel Flores-Kanter, Luciana Moretti, Roger Muñoz-Navarro, Paloma Ruíz-Rodríguez, Antonio Cano-Vindel, Leonardo Adrián Medrano

**Affiliations:** 1 Universidad Siglo 21, Córdoba, Argentina; 2 Faculty of Psychology, Universidad Nacional de Córdoba, Córdoba, Argentina; 3 Department of Personality, Assessment and Psychological Treatments, Faculty of Psychology, University of Valencia, Valencia, Spain; 4 Tres Cantos, Primary Care Center, Health Service of Madrid, Tres Cantos, Madrid, Spain; 5 Faculty of Psychology, Complutense University of Madrid, Madrid, Spain; 6 Department of Psychology, Pontificia Universidad Católica Madre y Maestra, Santiago de los Caballeros, Dominican Republic

**Keywords:** Depression, Mental health, Construct validity, Internal structure, CFA, ESEM

## Abstract

**Background:**

Depression is one of the most prevalent psychological disorders and the leading cause of disability worldwide. The development of effective strategies for the prevention and treatment of depression requires valid and reliable instruments capable of accurately assessing this condition. The present study examined the internal structure and measurement invariance of the Patient Health Questionnaire–9 (PHQ-9) in an Argentine population.

**Methods:**

Several measurement models proposed in the international literature were tested in a large Argentine sample (*N* = 5,950), comprising three independent subsamples: an online sample (*n* = 4,803), a clinical sample of individuals seeking psychological assistance (*n* = 98), and a random telephone sample (*n* = 1,049). PHQ-9 item responses were analyzed using Confirmatory Factor Analysis (CFA) and Exploratory Structural Equation Modeling (ESEM). In addition, measurement invariance was evaluated across gender, age groups, and administration formats.

**Results:**

The unidimensional model demonstrated good fit across most indices. Although two-factor models showed marginal improvements in fit, correlations between latent factors were consistently high (> 0.80). ESEM analyses indicated that these high correlations were not attributable to estimation bias arising from restrictive CFA specifications, further supporting a unidimensional structure. Moreover, the unidimensional model exhibited measurement invariance across gender, age groups, and administration formats.

**Conclusions:**

The findings support the use of the PHQ-9 to derive a global measure (e.g., sum score) of depressive symptom severity in the Argentine population, without systematic bias associated with demographic characteristics or mode of data collection. Further studies are warranted to replicate these results and extend validity evidence.

**Supplementary Information:**

The online version contains supplementary material available at 10.1186/s12888-026-07820-9.

## Introduction

Depression is one of the most significant global mental health problems, ranking among the most prevalent psychological disorders and representing the leading cause of disability worldwide [1]. It is estimated that more than 280 million individuals experience clinically significant depressive symptoms, placing depression among the main contributors to the global burden of disease. In Latin America and the Caribbean, the Pan American Health Organization has reported that approximately 22.4% of the population suffers from a mental disorder, with depression being the most prevalent, at an estimated rate of 5% [2]. In Argentina, recent studies indicate that depression is highly prevalent (8.7%) [3] and constitutes the leading cause of years lived with disability [4]. Beyond its prevalence, depression is characterized by a high likelihood of relapse and recurrence and is frequently associated with both psychological and medical comorbidities [5, 6]. In severe cases, it may lead to suicidal behaviors [7], underscoring the need to prioritize its detection, treatment, and prevention as a public health concern and to rely on brief, reliable, and culturally appropriate assessment tools for use across diverse settings.

Within this context, screening instruments play a critical role, particularly in primary health care and epidemiological research. The Patient Health Questionnaire-9 (PHQ-9) has become one of the most widely used instruments worldwide for assessing depressive symptoms due to its brevity, ease of administration, and strong psychometric properties [8–10]. The PHQ-9 assesses the frequency of core depressive symptoms over the past two weeks and is directly aligned with the diagnostic criteria for major depressive disorder established in the Diagnostic and Statistical Manual of Mental Disorders (DSM), as its items reflect the nine hallmark symptoms of this diagnosis [12]. The instrument has demonstrated adequate sensitivity (0.93) and specificity (0.85) [9], allowing for both continuous severity scoring and the application of clinically meaningful cutoff points. These characteristics support its utility for estimating depression severity and monitoring treatment response and outcomes [7, 11], thereby facilitating its use in a wide range of clinical and research contexts.

The PHQ-9 has been translated and validated across multiple languages and cultural contexts, with consistent evidence supporting reliability and validity in both clinical and non-clinical samples (Table 1). Notably, Bianchi, et al. [13] conducted an integrative psychometric analysis using data from 29 samples across seven countries (e.g., Germany and the United States) and five languages (e.g., German and English), yielding a combined sample of 58,272 participants—the largest study conducted to date. Their findings supported essential unidimensionality and demonstrated invariance across gender, age, and language. Nevertheless, extending the linguistic and cultural scope of the PHQ-9 remains necessary. This is particularly important in low- and middle-income countries, where the burden of mental disorders, including depression, is substantial and coexists with considerable linguistic and cultural heterogeneity [8]. Continued research on the cross-cultural and linguistic validation of the PHQ-9 is therefore warranted.

In Argentina, although the PHQ-9 has been used in local studies and applied settings, systematic psychometric evidence regarding its cultural adaptation and factorial structure remains limited and heterogeneous. Our review identified three previous studies assessing the psychometric properties of the instrument in Argentine samples [7, 14, 15]; however, none examined its internal structure through the comparison of alternative models using confirmatory factor analysis, nor did they employ more robust approaches such as *Exploratory Structural Equation Modeling* (ESEM). This lack of advanced factorial analyses limits current understanding of the instrument’s dimensionality in Argentina and highlights the need for evaluations consistent with contemporary methodological recommendations [16].

This limitation is particularly salient given the inconsistent findings reported in international studies (see Table 1). While some research supports a unidimensional structure of the PHQ-9 [10, 11, 17–21], other studies propose a two-factor solution distinguishing between “cognitive-affective” and “somatic” components. In these models, items 3, 4, and 5 (“sleep problems,” “fatigue,” and “changes in appetite”) typically load on the somatic factor, whereas items 1, 2, 6, 7, 8, and 9 (“anhedonia,” “depressed mood,” “feelings of worthlessness,” “concentration problems,” “slowness/agitation,” and “suicidal thoughts”) load on the cognitive-affective factor, although the relative contribution of individual items varies across studies [22–28]. Moreover, substantial heterogeneity has been observed in item allocation across two-factor models [29] (Table 2). Taken together, these inconsistencies underscore the need to clarify the internal structure of the PHQ-9, which is essential for the valid interpretation of its scores [13]. Ambiguous dimensionality complicates decisions regarding whether to rely on a single global severity score or to interpret distinct cognitive-affective and somatic subscores in clinical and applied research settings.

**Table 1:** Survey of studies on the factorial structure of the PHQ − 9

	One factor	Two factors
Huarcaya-Victoria, et al. [26]		X
Bi, et al. [30]		X
Peng, et al. [27]		X
Shin, et al. [22]		X
Leung, et al. [18]	X	
Kim and Lee [17]	X	
González-Blanch, et al. [11]	X	
Doi, et al. [24]	X	
Cassiani-Miranda, et al. [23]		X
Familiar, et al. [21]	X	
Petersen, et al. [28]		X
Ryan, et al. [20]	X	
Granillo, et al. [25]		X
Badeer, et al. [10]	X	
Merz, et al. [19]	X	
Donlan and Lee [31]	X	
Bianchi, et al. [13]	X	

**Table 2:** Items distribution in two factors models

	Models			
PHQ items	2a	2b	2c	2d
Item 1	NS (C-A)	NS (C-A)	NS (C-A)	NS (C-A)
Item 2	NS (C-A)	NS (C-A)	NS (C-A)	NS (C-A)
Item 3	S	S	S	S
Item 4	S	NS (C-A)	S	S
Item 5	S	S	S	S
Item 6	NS (C-A)	NS (C-A)	NS (C-A)	NS (C-A)
Item 7	S	S	NS (C-A)	NS (C-A)
Item 8	S	S	NS (C-A)	S
Item 9	NS (C-A)	NS (C-A)	NS (C-A)	NS (C-A)

Note: NS (C-A) = Non-somatic (cognitive/affective) factor; S = Somatic factor. Item 1 = anhedonia; Item 2 = dysphoria; Item 3 = sleep alterations; Item 4 = fatigue/loss of energy; Item 5 = appetite alterations; Item 6 = feelings of worthlessness; Item 7 = cognitive impairment; Item 8 = psychomotor alterations; Item 9 = thoughts of self-harm

Accordingly, the present study employs Confirmatory Factor Analysis (CFA) to compare the fit of a unidimensional model with that of four previously proposed two-factor models (see Table 2). In addition, a less restrictive modeling approach—ESEM—is applied. Unlike traditional CFA, ESEM allows for non-zero cross-loadings, making it particularly useful for evaluating potential parameter estimation biases associated with restrictive model specifications [32]. This consideration is especially relevant for the PHQ-9, as prior studies have reported high inter-factor correlations in two-factor models (ranging from 0.69 to 0.91) [24, 32–35]. Such correlations may reflect substantial substantive overlap between factors [36] or, alternatively, estimation artifacts resulting from constraining cross-loadings to zero in CFA models [32]. By relaxing these constraints, ESEM provides a more flexible framework for determining whether high factor correlations persist, thereby offering a more precise assessment of the PHQ-9’s dimensionality.

Finally, another aspect that has not yet been examined in the Argentine population is the measurement invariance of the PHQ-9’s dimensionality and structural properties. Establishing invariance is essential to ensure meaningful comparisons of scores across relevant groups [37]. Therefore, the present study also evaluates measurement invariance across gender, age, and administration format. Overall, this study aims to provide robust psychometric evidence supporting a valid and comparable interpretation of PHQ-9 scores in the Argentine context.

## Participants and procedure

### Participants

The total sample comprised 5,950 Argentine participants. The data were drawn from three independent samples who completed the PHQ-9 using different administration formats. One sample completed the questionnaire online (*n* = 4,803), a second sample completed it via telephone administration (*n* = 1,049), and a third sample completed the PHQ-9 in the context of a clinical interview (*n* = 98).

Participants were recruited through an open online sampling approach [38]. Data collection was conducted using a web-based questionnaire hosted on the Google Forms platform, which was disseminated via the social media network Facebook. Prior to completing the survey, all respondents provided informed consent by selecting an “I accept” option that granted access to the questionnaire. Eligibility was limited to individuals aged 18 years or older, with no additional exclusion criteria imposed. Submission of the survey was restricted until all questionnaire items had been fully completed. No specific measures were employed to assess or regulate response quality, such as procedures to identify inattentive or careless responding.

The telephone sample consisted of 1,049 participants selected through a random telephone sampling procedure. To this end, the geographical areas to be included and their corresponding telephone area codes were first defined. The sample comprised employed individuals from the following cities: Buenos Aires (Capital Federal) (30.9%), Comodoro Rivadavia (11.0%), Córdoba (17.3%), Corrientes (8.3%), Mendoza (10.1%), Rosario (14.6%), and San Miguel de Tucumán (7.8%). Verbal informed consent was obtained from all participants prior to participation. The only inclusion criterion was being 18 years of age or older, and no exclusion criteria were applied.

Finally, the clinical sample was composed of individuals seeking psychological assistance. Participants were recruited through the Emotional Well-being Assessment and Promotion Program (Psicap), which is part of the outreach services of the Faculty of Psychology at the National University of Córdoba, Argentina. Individuals voluntarily requested an appointment via email or telephone, at which point an assessment session was scheduled. During the in-person meeting, participants completed the PHQ-9 after providing written informed consent. As with the other samples, the sole inclusion criterion was being 18 years of age or older, and no exclusion criteria were applied.

The study was conducted in accordance with internationally recognized ethical standards for research involving human participants [39], and prior approval of the research protocol was obtained from the Ethics Committee of the Research Secretariat of Universidad Siglo 21. Before taking part, all individuals received comprehensive information regarding the study aims, the anonymous treatment of their data, and the voluntary nature of their involvement. Participants were further informed that the study entailed no risk or potential harm and that they could discontinue their participation at any moment without any negative consequences. No monetary or non-monetary incentives were offered in exchange for participation.

### Instrument

*Patient Health Questionnaire-9* (PHQ-9) [40]. The PHQ-9 is a subscale of the Patient Health Questionnaire that comprises nine items corresponding to the diagnostic criteria for major depressive disorder as defined in the Diagnostic and Statistical Manual of Mental Disorders (DSM-IV). The scale assesses the presence of the following symptoms over the preceding two weeks: (a) depressed mood, (b) anhedonia, (c) sleep disturbances, (d) fatigue, (e) changes in appetite or weight, (f) feelings of guilt or worthlessness, (g) difficulty concentrating, (h) psychomotor retardation or agitation, and (i) suicidal thoughts.

Items are rated on a four-point Likert scale ranging from 0 to 3: 0 (not at all), 1 (several days), 2 (more than half the days), and 3 (nearly every day). The Spanish version of the scale was administered [41], which has demonstrated good internal consistency (McDonald’s ω = 0.89), as well as adequate sensitivity (88%) and specificity (80%) [42]. In the present study, the PHQ-9 showed excellent internal consistency, as indicated by the factor loadings of the unidimensional model (ω_*Total Sample*_ = 0.927; 95% CI [0.924, 0.930]).

### Data analysis procedure

PHQ-9 item responses were analyzed using both CFA and ESEM. Given the categorical nature of the item indicators, the weighted least squares estimator with mean- and variance-adjusted standard errors (WLSMV) was employed, as this method is widely recommended for models involving ordinal categorical variables [43]. For the ESEM models, factors were rotated using Geomin and Oblimin rotation methods [44, 45]. Factor loadings of 0.40, 0.55, and 0.70 were interpreted as low, moderate, and high, respectively [46].

Model fit was assessed through a set of five complementary fit indicators: the χ²-to-degrees-of-freedom ratio (χ²/df), the Comparative Fit Index (CFI), the Tucker–Lewis Index (TLI), the Root Mean Square Error of Approximation (RMSEA), and the Standardized Root Mean Square Residual (SRMR). Ratios of χ²/df equal to or below 2–3 were taken as evidence of an acceptable fit [47]. CFI and TLI values of 0.90 or above were regarded as indicative of adequate model fit, while values reaching 0.95 or higher were considered to represent excellent fit. For the RMSEA, values lower than 0.08 were interpreted as reflecting reasonable fit and those below 0.05 as indicating close fit [47–49]. With respect to the SRMR, values not exceeding 0.08 were viewed as suggesting good correspondence between the model and the observed data [50]. Additionally, 90% confidence intervals were computed and reported for the RMSEA. It is important to acknowledge that fit statistics may be affected by elements other than true model misspecification, such as incidental parameters and model complexity [46, 51]. Therefore, these indices should be interpreted in combination rather than as rigid cutoff thresholds, and conclusions regarding model fit should be drawn with due caution [52].

Measurement invariance analyses were conducted across gender, age groups, and sample type following three sequential levels of invariance testing [37, 53]: (a) configural invariance, (b) metric (weak) invariance, and (c) scalar (strong) invariance. Configural invariance assumes that the same factor structure (specifically, the number of factors and the pattern of relationships between items and factors) is maintained across groups. Metric invariance further constrains factor loadings to be equal across groups, while scalar invariance additionally requires item thresholds to be invariant. A given level of invariance was considered supported when the fit of the more constrained model, relative to the configural model, did not decrease by more than 0.01 in the CFI or increase by more than 0.015 in the RMSEA [54]. Delta parameterization was applied in all measurement invariance models.

Descriptive analyses were conducted in R (version 4.3.2; 2023-10-31 ucrt) using RStudio [55]. These analyses relied on base R functions and packages (e.g., the cor and var functions from the stats package). All CFA, ESEM, and measurement invariance analyses were performed using Mplus (version 8.6) [56].

## Results

### Descriptives and correlations

First, descriptive analyses were conducted. The majority of the total sample corresponded to the online administration format (80.72%), followed by the telephone sample (17.63%) and the clinical sample (1.64%). Regarding sociodemographic characteristics, considering all samples combined, 65.09% of participants were women, and the largest proportion of respondents belonged to the young adult age group (80.75%).

Missing data were observed only in the telephone sample, as participants were allowed to choose not to answer certain questions in this format. Nevertheless, the proportion of complete data was high across all PHQ-9 items, ranging from 98.76% to 99.61%. Table 3 presents the Pearson correlations among the nine PHQ-9 items. All correlations were positive, with values ranging from 0.341 to 0.623. In addition, Table 3 reports the means, standard deviations, and variances for each item.

**Table 3:** Correlation and descriptive results

	p1	p2	p3	p4	p5	p6	p7	p8	p9
p1	1.08								
p2	0.62	1.22							
p3	0.40	0.50	1.29						
p4	0.51	0.62	0.56	1.01					
p5	0.40	0.47	0.49	0.54	1.17				
p6	0.50	0.66	0.46	0.55	0.45	1.41			
p7	0.45	0.53	0.43	0.53	0.44	0.55	1.24		
p8	0.36	0.44	0.36	0.41	0.40	0.44	0.50	1.15	
p9	0.42	0.56	0.34	0.42	0.34	0.60	0.43	0.39	1.31
Mean	1.53	1.64	1.81	1.95	1.80	1.49	1.40	1.22	0.87
SD	1.04	1.10	1.14	1.01	1.08	1.19	1.11	1.07	1.14

Note. SD = standard deviation. p1 to p9 = PHQ-9 items: Item 1 = anhedonia; Item 2 = dysphoria; Item 3 = sleep alterations; Item 4 = fatigue/loss of energy; Item 5 = appetite alterations; Item 6 = feelings of worthlessness; Item 7 = cognitive impairment; Item 8 = psychomotor alterations; Item 9 = thoughts of self-harm. On the diagonal are the variances of the respective items (highlighted in light gray). Below the diagonal are the zero-order correlation coefficients. All correlations are statistically significant (*p* < .001)

### Confirmatory Factor Analysis and ESEM

The first step consisted of evaluating the fit of the different measurement models proposed for the PHQ-9 (see Table 4). The unidimensional model demonstrated optimal fit across most of the global fit indices considered. Although the two-factor models showed some improvement in fit indices, the estimated correlations between the latent factors were consistently high, exceeding 0.80 in all cases (see Table 5). In Table S1 of the Supplementary Material, the confidence intervals for the factor loadings and the proportion of explained variance derived from the unidimensional model are reported.

**Table 4:** Fit statistics for the CFA estimated models and ESEM

Model							
CFA	χ^2^	*df*	χ^2^/*df*	TLI	CFI	SRMR	RMSEA (90% CI)
One-factor	1847.30	27	68.42	0.962	0.972	0.035	0.102 (0.103, 0.111)
2a	952.89	26	36.65	0.980	0.986	0.025	0.077 (0.073, 0.082)
2b	1564.80	26	60.18	0.967	0.976	0.032	0.100 (0.096, 0.104)
2c	1128.86	26	43.42	0.976	0.983	0.027	0.084 (0.080, 0.089)
2d	1341.95	26	51.61	0.972	0.980	0.030	0.092 (0.088, 0.096)
ESEM	χ^2^	*df*	χ^2^/*df*	TLI	CFI	SRMR	RMSEA (90% CI)
Two-factor	815.735	19	42.93	0.976	0.988	0.021	0.084 (0.079, 0.089)

Note. 2a to 2d = two-factors models (see Table 2); ESEM = Exploratory Structural Equation Modeling; CFA = Confirmatory Factor Analysis; χ^2^ = chi-square; *df* = degrees of freedom; CFI = comparative fit index; SRMR = standardized root mean square residual; RMSEA = root mean square error of approximation

**Table 5:** Factor loadings derived from model estimation and interfactor correlations

				CFA Models					
	One-fact.	2a		2b		2c		2d	
Items		S	N-S	S	N-S	S	N-S	S	N-S
Item 1	**0.723**	*0.00*	**0.741**	*0.00*	**0.728**	*0.00*	**0.731**	*0.00*	**0.732**
Item 2	**0.872**	*0.00*	**0.898**	*0.00*	**0.878**	*0.00*	**0.884**	*0.00*	**0.885**
Item 3	**0.707**	**0.727**	*0.00*	**0.734**	*0.00*	**0.745**	*0.00*	**0.732**	*0.00*
Item 4	**0.824**	**0.857**	*0.00*	*0.00*	**0.834**	**0.885**	*0.00*	**0.864**	*0.00*
Item 5	**0.690**	**0.709**	*0.00*	**0.715**	*0.00*	**0.727**	*0.00*	**0.714**	*0.00*
Item 6	**0.839**	*0.00*	**0.861**	*0.00*	**0.846**	*0.00*	**0.848**	*0.00*	**0.849**
Item 7	**0.745**	**0.772**	*0.00*	**0.77**	*0.00*	*0.00*	**0.755**	*0.00*	**0.757**
Item 8	**0.636**	**0.656**	*0.00*	**0.658**	*0.00*	*0.00*	**0.644**	**0.661**	*0.00*
Item 9	**0.775**	*0.00*	**0.792**	*0.00*	**0.781**	*0.00*	**0.782**	*0.00*	**0.783**
S	-	1.00	-	1.00	-	1.00	-	1.00	-
N-S	-	0.885	1.00	0.922	1.00	0.880	1.00	0.904	1.00
		**ESEM**							
		**Two-factor**							
		**S**	**N-S**						
Item 1	-	0.143	**0.608**	-	-	-	-	-	-
Item 2	-	0.122	**0.683**	-	-	-	-	-	-
Item 3	-	**0.760**	*0.000*	-	-	-	-	-	-
Item 4	-	**0.682**	0.201	-	-	-	-	-	-
Item 5	-	**0.744**	*− 0.002*	-	-	-	-	-	-
Item 6	-	0.008	**0.855**	-	-	-	-	-	-
Item 7	-	**0.415**	**0.373**	-	-	-	-	-	-
Item 8	-	**0.368**	**0.305**	-	-	-	-	-	-
Item 9	-	− 0.234	**1.01**	-	-	-	-	-	-
S	-	1.00	-	-	-	-	-	-	-
N-S	-	0.806	1.00	-	-	-	-	-	-

Note. One-fact. = one factor model; CFA = Confirmatory Factor Analysis; ESEM = Exploratory Structural Equation Modeling. The geomin rotation is shown for the ESEM solution. Factor loadings ≥ 0.30 in absolute value are bolded and highlighted in grey. Cross-loadings fixed to zero appear in italics. *p* < .05 for all factor loadings, factor correlations, except those underlined. S = Somatic Factor; N-S: No Somatic. Item 1 = anhedonia; Item 2 = dysphoria; Item 3 = sleep alterations; Item 4 = fatigue/loss of energy; Item 5 = appetite alterations; Item 6 = feelings of worthlessness; Item 7 = cognitive impairment; Item 8 = psychomotor alterations; Item 9 = thoughts of self-harm

To examine whether the high inter-factor correlations observed in the CFA models were attributable to estimation bias resulting from restrictive model specifications, ESEM analyses were subsequently conducted. The results indicated that factor correlations remained consistently high and did not change substantially (i.e., > 0.80; see Table 5). Similar results were obtained using both Oblimin and Geomin rotation methods; for simplicity, only the results derived from the Oblimin rotation are presented in the table.

Overall, the factor analytic results provide evidence in support of an essentially unidimensional structure of the PHQ-9 in the Argentine sample. As shown in Table 4 and 5, the one-factor CFA model demonstrated adequate fit according to most global fit indices considered (i.e., TLI, CFI, and SRMR), with all factor loadings being statistically significant and of substantial magnitude. By contrast, the two-factor solution did not yield a meaningful improvement in model fit, and the correlation between the two latent factors remained high, indicating limited discriminant validity between the proposed dimensions.

**Table 6:** Factorial invariance analyses across sex, age groups (young adults, middle-aged adults, and older adults), and administration format (on-line vs. telephone)

	Overall Model Fit					Change in Model Fit				
	χ^2^	df	CFI	SRMR	RMSEA	Δχ^2^	Δdf	ΔCFI	ΔSRMR	ΔRMSEA
Sex										
MI1. Configural	1841.54	54	0.971	0.036	0.106					
MI2. Metric (FL)	1779.56	62	0.972	0.037	0.097	52.18	8	0.001	0.001	− 0.009
MI3. Scalar (FL, Th)	1803.81	79	0.972	0.038	0.086	256.85	25	0.001	0.002	− 0.020
Age Groups										
MI1. Configural	1961.46	81	0.965	0.038	0.108					
MI2. Metric (FL)	1893.07	97	0.967	0.039	0.097	54.22	16	0.002	0.001	− 0.011
MI3. Scalar (FL, Th)	1750.87	131	0.970	0.040	0.079	195.23	50	0.005	0.002	− 0.029
Type of sample										
MI1. Configural	1687.15	54	0.962	0.044	0.102					
MI2. Metric (FL)	1655.01	62	0.963	0.048	0.094	121.94	8	0.001	0.004	− 0.008
MI3. Scalar (FL, Th)	2131.37	79	0.953	0.060	0.094	661.97	25	− 0.009	0.012	− 0.008

Note. Group sizes: (a) sex: female = 3870, male = 2075; (b) age: 18–39 = 4802, 40–49 = 627, ≥ 50 = 517; (c) phone database = 1046, base online = 4803. χ^2^ = chi-square; *df* = degrees of freedom; CFI = comparative fit index; SRMR = standardized root mean square residual; RMSEA = root mean square error of approximation; MI = measurement invariance; FL = factor loadings; Th = thresholds; The parameters constrained to be equal across groups are shown in the parentheses next to the invariance models. The chi-square difference tests between nested models were conducted using Mplus’ DIFFTEST option. *p* < .001 for all chi-square tests. Changes in model fit were computed against the configural model

It should be noted that neither the one-factor nor the two-factor models achieved acceptable fit according to the χ²/df and RMSEA indices. To further examine model performance, the two-factor structure was also tested using ESEM; however, this approach resulted in only marginal differences in fit and did not reveal substantially different loading patterns. Taken together, these findings reinforce the interpretation of the PHQ-9 as essentially unidimensional in this context and support the use of a single global severity score for the assessment of depressive symptoms.

### Measurement invariance analysis

Finally, measurement invariance analyses were conducted across gender, age groups, and administration format (online vs. telephone). Age groups were defined according to the criteria proposed by Martín Ruiz [57]: young adults (18–40 years; *n* = 4,805; 80.76%), middle-aged adults (41–50 years; *n* = 627; 10.54%), and older adults (> 50 years; *n* = 518; 8.70%).

The observed changes in CFI and RMSEA across the successive invariance models indicated that measurement invariance was supported across gender, age groups, and administration format (Table 6). Specifically, no meaningful deterioration in model fit was observed when equality constraints were imposed across groups, supporting the equivalence of the PHQ-9 measurement properties across these conditions.

## Discussion

The present study aimed to evaluate the factorial structure and measurement invariance of the PHQ-9 in an Argentine population using robust psychometric approaches. To this end, alternative factorial models were compared using CFA and ESEM, and measurement invariance was examined across gender, age groups, and administration formats. Overall, the findings provide consistent evidence supporting an essentially unidimensional structure of the PHQ-9 and demonstrate measurement invariance across key sociodemographic and methodological variables, thereby reinforcing the validity and comparability evidence of PHQ-9 scores in the Argentinean context.

The results consistently support an essentially unidimensional measurement model for the PHQ-9. This conclusion is substantiated by the adequate global and local fit of the one-factor model, as well as by the high correlations observed between latent factors when two-factor solutions were specified [36]. Because such high correlations may sometimes arise as artifacts of restrictive CFA specifications, ESEM models were estimated to relax cross-loading constraints [32]. Notably, the application of ESEM did not yield a meaningful reduction in factor correlations, which remained above 0.80. This pattern suggests that the observed correlations are unlikely to reflect estimation bias and instead indicate substantial overlap between the proposed dimensions, favoring a unidimensional representation of depressive symptom severity.

Consistent with these findings, dimensionality evidence derived from PHQ-9 scores points to the presence of a general factor accounting for a substantial proportion of the common variance among items [13]. Although prior research reports considerable heterogeneity regarding the internal structure of the PHQ-9, most studies also support an adequate fit of the unidimensional model [13, 19, 21,58,59–64]. Moreover, when two-factor models are proposed, latent factor correlations are typically high, ranging from 0.69 to 0.91 [24, 33–35]. Even in studies favoring a two-factor solution after model comparisons, the one-factor model generally achieves acceptable fit according to commonly applied criteria [24, 34, 65]. Taken together, these findings—along with the present results—support the use of the PHQ-9 as a measure of overall depression severity [58], rather than as a multidimensional instrument yielding distinct subscale scores.

Nevertheless, the strength of the PHQ-9 in producing a global severity score also entails certain limitations. Specifically, the instrument does not allow for a fine-grained assessment of structural differentiation among depressive symptoms. Substantial evidence indicates that depressive symptoms are not interchangeable but instead represent structurally distinct components that may show different patterns of association with one another and with external criteria [66–68]. This symptom-level heterogeneity may help explain why some fit indices obtained in the present study—particularly χ², χ²/df, and RMSEA—did not reach conventional thresholds for acceptable fit. Indeed, inspection of the proportion of variance explained by the general factor (Table S1) indicates that a nontrivial amount of item variance (approximately 24% to 60%) remains unexplained, reflecting unique variance.

This residual variance may also account for the relatively favorable performance of two-factor models, as subsets of symptoms (e.g., somatic versus cognitive-affective symptoms) may be more strongly interrelated within an otherwise heterogeneous symptom set. However, it is important to emphasize that neither the one-factor nor the two-factor models achieved acceptable fit according to χ², χ²/df and RMSEA. As noted in previous methodological work, these indices are sensitive to factors unrelated to substantive model misfit, including sample size and incidental model parameters [46, 51], and therefore should not be interpreted as strict decision rules [52]. When considered jointly with other fit indices and the broader pattern of results, the evidence presented here supports the use of a single global depression severity score derived from the PHQ-9. If the research or clinical objective is instead to capture the structural differentiation of depressive symptoms, alternative measurement instruments or modeling approaches should be considered [69].

It is also important to note that the PHQ-9 was specifically designed to operationalize the nine diagnostic criteria for major depressive disorder as defined in the Diagnostic and Statistical Manual of Mental Disorders [70]. This is particularly relevant in light of evidence identifying a much broader pool of depressive symptoms—up to 52 distinct symptoms—across commonly used depression scales [71], many of which are not represented in the PHQ-9. Consequently, the choice of measurement strategy should be guided by the specific aims of assessment. In the present study, our findings support the defensibility of using a global PHQ-9 score to assess depression severity as defined by DSM-based criteria, rather than to capture the full phenomenological complexity of depressive symptomatology.

Finally, the present study contributes novel evidence regarding an aspect that has received relatively limited attention in prior research [13]: the measurement invariance of the PHQ-9 across gender, age groups, and administration formats. The results indicate that the factorial structure of the PHQ-9 is invariant across all tested conditions, suggesting that observed differences in PHQ-9 scores are unlikely to be attributable to measurement bias. With respect to administration format, these findings extend recent evidence supporting the validity of PHQ-9 scores obtained via online and telephone assessments [72], an issue of particular relevance given the increasing reliance on remote data collection methods—a trend further accelerated by the COVID-19 pandemic. Consistent with previous studies [13], invariance across gender and age was also supported. Collectively, these results indicate that inferences regarding depressive severity based on PHQ-9 scores are comparable across administration modes and demographic groups within the Argentine context.

### Constraints on generality and limitations

Several limitations should be considered when interpreting the findings of the present study. First, the sociodemographic composition of the sample may constrain the generalizability of the results. Across the three subsamples, women were overrepresented (65.09%), and the majority of participants were classified as young adults (80.75%). In addition, no information was collected on other relevant dimensions of diversity, such as ethnicity or socioeconomic status. Although Argentina does not fall within the WEIRD (Western, Educated, Industrialized, Rich, and Democratic) classification, the predominance of urban and online respondents may reflect similar characteristics, including higher educational attainment and greater access to digital resources. Consequently, caution is warranted when extending these findings to underrepresented demographic groups within the Argentine population.

A related consideration applies to the telephone subsample, which consisted exclusively of employed individuals from selected urban areas. While this limits full representativeness, a notable strength of the present study is its broad geographical coverage, which included multiple cities across different regions of the country, including less densely populated areas (e.g., Comodoro Rivadavia, Corrientes, and San Miguel de Tucumán). This regional diversity partially offsets representativeness concerns and strengthens the robustness of the overall evidence compared to prior studies focused solely on Buenos Aires. Future research should aim to include more diverse and representative samples in terms of age, gender identity, socioeconomic background, and geographic context.

From a methodological perspective, several additional limitations merit consideration. First, criterion validity was not examined using external measures, which limits conclusions regarding the convergent or predictive validity of PHQ-9 scores. Second, the stability of the results over time was not assessed. Future studies could address this limitation by incorporating test–retest reliability analyses, bootstrapping procedures, or cross-validation across independent samples. Moreover, the cross-sectional design precludes the evaluation of longitudinal measurement invariance, which is particularly relevant for supporting the use of the PHQ-9 in monitoring change over time (e.g., treatment response).

Additional limitations relate to methodological decisions commonly encountered in psychometric research. Specifically, the use of conventional cutoff values for global fit indices may be suboptimal, as growing evidence supports the use of dynamic fit index cutoffs tailored to specific models and data conditions [73]. Likewise, unbalanced group sizes in measurement invariance analyses may reduce sensitivity to detect non-invariance [74]. Future studies should examine the replicability of the present findings using dynamic cutoff criteria and more balanced group designs. Finally, no specific procedures were implemented to assess or control response quality in the online sample, an issue that has been increasingly recognized as relevant in large-scale survey research [75, 76] and should be addressed in future investigations.

## Conclusion

The findings of the present study provide robust evidence supporting an essentially unidimensional factor structure of the PHQ-9 and demonstrate measurement invariance across sex, age groups, and administration formats in an Argentine population. Together, these results support the use of the PHQ-9 as a reliable instrument for deriving a global measure of depressive symptom severity, without systematic bias attributable to demographic characteristics or mode of data collection.

Importantly, support for a unidimensional measurement model should be understood as a conclusion regarding the psychometric properties of the PHQ-9 rather than as evidence that depression itself represents a single latent construct. Depression remains a heterogeneous and multifaceted phenomenon. The present findings simply indicate that, within this context, PHQ-9 item responses can be meaningfully summarized into a general severity score for measurement purposes. Continued research is warranted to replicate these findings in other Argentine samples, to apply alternative psychometric approaches, and to expand validity evidence across additional phases of construct validation.

## Electronic Supplementary Material

Below is the link to the electronic supplementary material.

Supplementary Material 1

## Data Availability

The datasets used and/or analysed during the current study are available from the corresponding author on reasonable request.
