# Supplementary material for: Internal structure and factorial invariance of the Patient Health Questionnaire −9 (PHQ-9) in a large Argentinean sample
Source: BMC Psychiatry. 2026 Jan 28;26:196. doi: 10.1186/s12888-026-07820-9 (PMC12924556; doi:10.1186/s12888-026-07820-9)
Supplement: Supplementary file 1 — Supplementary Material 1 [file 12888_2026_7820_MOESM1_ESM.docx]

**Supplementary Material**

Table S1.

*Factor loadings from one-factor (CFA) model, 95% confidence intervals, and R^2^.*

| **Item** | **λ** | **λ 95% CI** | **R^2^** |
| --- | --- | --- | --- |
| Item 1 | .723 | .706; .740 | .522 |
| Item 2 | .872 | .861; .882 | .760 |
| Item 3 | .707 | .688; .725 | .499 |
| Item 4 | .824 | .812; .837 | .680 |
| Item 5 | .690 | .671; .708 | .475 |
| Item 6 | .839 | .828; .851 | .704 |
| Item 7 | .745 | .730; .762 | .555 |
| Item 8 | .636 | .614; .657 | .405 |
| Item 9 | .775 | .759; .790 | .601 |

*Note*. λ = Factor loadings; λ 95% CI = 95% confidence intervals for the estimated factor loading.
